# Familiarity of Physicians and Nurses with Different Aspects of Oxygen Therapy; a Brief Report

**Published:** 2017-01-11

**Authors:** Reza Goharani, MirMohammad Miri, Mehran Kouchek, Mohammad Sistanizad

**Affiliations:** 1 Department of Critical Care, Imam Hossein Hospital, Shahid Beheshti University of Medical Sciences, Tehran, Iran.; 2 Department of Clinical Pharmacy, Faculty of Pharmacy, Shahid Beheshti University of Medical Sciences, Tehran, Iran.; 3 Department of Pharmaceutical Care Unit, Imam Hossein Hospital, Shahid Beheshti University of Medical Sciences, Tehran, Iran.

**Keywords:** Oxygen, oxygen inhalation therapy, knowledge, adverse effects, physicians, nurses

## Abstract

**Introduction:**

Oxygen is a drug and physician and nurses should be familiar with the effects and potential risks of oxygen therapy. The current study aimed to assess familiarity of physicians and nurses with various aspects of oxygen therapy.

**Method:**

In this cross sectional study, the familiarity of physicians and nurses with various aspects of oxygen therapy in a teaching hospital was evaluated using a validated questionnaire. The collected data were analyzed using SPSS 21 software.

**Results:**

57 physicians and 79 nurses returned the completed questionnaire (response rate 97.1%). Mean clinical work experience of participants was 6.9±5.7 (1–15) years.

98.2% of physicians believed that oxygen therapy can be associated with risk and should be recorded in the patient's medical file. These measures were 92.4% and 98.2% for nurses. 38 (27.9%) participants correctly pointed out the reasons for oxygen therapy. Regarding necessary measurements and monitoring for oxygen therapy, 49 (86%) physicians and 65 (82.3%) nurses chose the correct answer. In addition, regarding necessity of blood gas analysis during oxygen therapy, 44 (77.2%) physicians and 55 (69.6%) nurses chose the correct answer.

**Conclusion:**

The findings showed that the familiarity level of participants with some aspects of O_2_ therapy such as its indications, necessary measurements and monitoring during therapy, and identifying delivery devices was fair to weak (<80%).

## Introduction

Oxygen is one of the most common drugs used in secondary care in hospital. Oxygen is an essential component of resuscitation, acute medical care, basic life support, anesthesia and postoperative care. When oxygen is used appropriately, it can save lives. On the other hand, any errors in oxygen therapy can worsen a patient’s condition and can even be life-threatening (1). Nowadays, the benefits and potential complications of oxygen therapy are well known; however, oxygen therapy is often done without special attention and sufficient knowledge (-). There are several guidelines and protocols for appropriate oxygen therapy (2, 6). It has been shown that use of protocols for oxygen therapy in hospital wards can be very helpful in reducing errors during this process (-). Previous studies have evaluated the use of oxygen in hospitals (10, 11). These studies reveal that doctors often prescribe oxygen improperly and without adequate supervision. Modified charts for prescribing oxygen and related requirements have been proposed (2, 11). Dodd et al. have shown that introduction of a specific prescription chart for oxygen improves the quality of its prescription by junior doctors from 55% to 91%. However, these recommendations had a positive effect on patient care only if health professionals had proper information and sufficient understanding of oxygen therapy and its correct administration (12). 

To ensure the safe and effective oxygen delivery, flow rate, dose, devices, time, and method of monitoring should be given special attention and doctors and nurses should be familiar with the effects and potential risks of oxygen therapy.

Based on the above-mentioned, this study aimed to assess familiarity of physicians and nurses with various aspects of oxygen therapy.

## Methods

***Study design:***

This cross sectional study was conducted in a teaching hospital affiliated to Shahid Beheshti University of Medical Sciences, Tehran, Iran, in 2014. The familiarity of physicians and nurses with various aspects of oxygen therapy was evaluated using a predesigned questionnaire. After a full explanation about the study design, written informed consent was obtained from all nurses and physicians. All information about the participants was kept confidential. The study protocol was approved by the ethics committee of Shahid Beheshti University of Medical Sciences.

***Participants***

60 physicians and 80 nurses working in different hospital wards were enrolled. All participants had some experiences in oxygen therapy. It was assumed that all participants had the basic skills and training about oxygen therapy. Questionnaires were anonymous and 20 minutes were given to each participant to complete the questionnaire without conferring.

***Questionnaire***

Internet search for international and local oxygen delivery guidelines was done and the most common and appropriate ones were used for designing a questionnaire (13, 14). Two consultant respiratory physicians, independently evaluated and confirmed the questionnaire items and responses. It was expected that questions about parameters in oxygen therapy would assess topics that all doctors and nurses had undergone in education and training courses.

In the first part of the questionnaire, participants were asked to identify oxygen delivery devices and choose their correct names. Then in the second part, a variety of questions about how to prescribe and deliver oxygen, and reasons for oxygen prescription were asked.

The familiarity rate was categorized into five groups based on Likert scale: ≥ 90% as excellent, 80-90% good, 70-80% fair, 60-70% weak and < 60% poor.

***Statistical analysis***

Data from the completed questionnaires were extracted and analyzed using SPSS version 17. To describe the data, frequency, percentage, mean, standard deviation, median, and interquartile range (IQR) were used.

## Results

***Baseline characteristics***

60 physicians and 80 nurses were studied. The response rate was 97.1%, and a total of 136 participants [57 physicians (54.4% female) and 79 nurses (70.9% female)] were involved. Average work experience of participants was 6.9 ± 5.7 years (1-14). 70 (51.5%) participants stated that there was a protocol to prescribe oxygen in their ward. The usual method of oxygen delivery in hospital wards were nasal cannula (58.1%) and simple mask (33.1%), respectively. Table 1 shows the usage percentage of different O_2_ delivery devices in the studied hospital.

**Table 1:** The most often used oxygen delivery devices in Imam Hossein hospital

**Devices **	**Number (%)**
**Nasal cannula**	79 (58.1)
**Simple face mask**	45 (33.1)
**Non-Rebreathing face mask with reservoir bag**	6 (4.4)
**Venturi mask**	6 (4.4)

**Table 2:** Familiarity of participants with different oxygen delivery devices

**Devices **	**Nurses **	**Doctors **
**Nasal specs/prongs/cannula**	79 (100)	54 (94.7)
**Bag mask ventilation**	78 (98.7)	57 (100)
**Venturi mask**	76 (96.2)	50 (87.7)
**Rebreathing face mask**	41 (51.9)	40 (70.2)
**Non-rebreathing face mask with reservoir bag**	41 (51.9)	45 (78.9)

Data were presented as number and percentage.

***Knowledge***

98.2% of physicians believed that oxygen therapy can be associated with risk and should be recorded in the patient's medical file. These measures were 92.4% and 98.2% for nurses. Table 2 shows the familiarity percentage of participants with different oxygen delivery devices. 38 (27.9%) participants correctly pointed out the reasons for oxygen therapy. Regarding necessary measurements and monitoring for oxygen therapy, 49 (86%) physicians and 65 (82.3%) nurses chose the correct answer. In addition, regarding necessity of blood gas analysis during oxygen therapy, 44 (77.2%) physicians and 55 (69.6%) nurses chose the correct answer.

## Discussion

The findings showed that the familiarity level of participants with some aspects of O_2_ therapy such as its indications, necessary measurements and monitoring during therapy, and identifying delivery devices was fair to weak (<80%).

Previous studies had evaluated the use of oxygen at hospitals in other countries (10, 11). In a study conducted in 2006 by Ganeshan et al., knowledge of 53 nurses and 40 doctors that worked in intensive care unit of the General Hospital in UK and were active in oxygen prescription, was evaluated. 25% of the physicians and 50% of the nurses could not prescribe the right dose and method of oxygen therapy in cardiorespiratory arrest cases. They concluded that doctors and nurses did not have sufficient knowledge and understanding of oxygen therapy (9).

In a similar study, Brokalaki et al. assessed the knowledge of oxygen therapy in seven hospitals in a major city of Greece, in 2004. The questionnaire was completed by 105 head-nurses. The results showed that training programs, protocols and guidelines should be mandatory to ensure proper use of oxygen therapy by nursing personnel (14).

In our study, approximately half of participants said that there is no protocol to prescribe oxygen in their wards, despite the existence of several international guidelines for proper oxygen therapy (2, 6).

However, only 51.9 to 78.9% of the nurses and doctors recognized O_2_ delivery devices such as non-rebreathing mask with reservoir bag, which is consistent with Ganeshan et al. results (13). 

In the present study, only 38 participants (27.9%) correctly pointed out the indications for oxygen therapy, namely decreased level of consciousness, chest pain, respiratory distress, seizure, severe respiratory infections and sepsis.

98.2% of physicians and 92.4% of nurses believed that oxygen therapy can be associated with risk. Any error in oxygen therapy could lead to worsening of the patient's status and can even be life-threatening (1).

Although oxygen is used for the treatment of hypoxia, it can be deadly and should be considered as a drug (2). In our study, 33 (57.98%) physicians and 47 (95.5%) nurses believed that oxygen is a drug. In another study, it was shown that 59% of a hospital's head nurses believe that oxygen is a drug and should be administered with prescription, while 41% believe that the oxygen is a gas that improves patients’ breathing (14).

This study reveals that the knowledge of doctors and nurses on how to correctly use oxygen is fair to weak (in some aspects) and this could have a harmful effect on their performance. 

It is clear that more emphasis on training in oxygen therapy is necessary during basic training courses for doctors and nurses; and constant and dynamic monitoring on personnel’s learning and performance should be applied. 

Therefore, more consideration and further theoretical and practical training courses in this field seems to be necessary.

Small sample size and failure to evaluate the attitude and practice of participants are among the limitations of the present study and it is recommended to conduct more studies in this regard.

## Conclusion:

The findings showed that the familiarity level of participants with some aspects of O_2_ therapy such as its indications, necessary measurements and monitoring during therapy, and identifying delivery devices was fair to weak (<80%). Therefore, more consideration and further theoretical and practical training courses in this field seems to be necessary.
